# Supplementary material for: Complexome profiling of the Chlamydomonas psb28 mutant reveals TEF5 as an early PSII assembly factor
Source: Plant Cell. 2025 Mar 17;37(6):koaf055. doi: 10.1093/plcell/koaf055 (PMC12164590; doi:10.1093/plcell/koaf055)
Supplement: koaf055_Supplementary_Data [file koaf055_supplementary_data.zip › psb28_interactive.zip!/plots/intensity/qualityPlots/peptides/_Cre01.g024350.t1.2.html]

### Cre01.g024350.t1.2

Trivial name:   
SeqCoverage [median]: 12.40  
Mass: 69.97 kDa  
  
 

| Sequence | MissedCleavage | Mass [Da] | wtIntensity | muIntensity | Score |
| --- | --- | --- | --- | --- | --- |
| ASLMFVR | 0 | 822.44 | 944.28 | 1523.09 | 125.08 |
| FTYSVK | 0 | 743.39 | 0.00 | 4034.12 | 77.50 |
| HKLLGMGEVVDANR | 1 | 1537.80 | 0.00 | 926.20 | 66.99 |
| LFKPGGVTEYK | 1 | 1237.67 | 2447.87 | 8439.86 | 91.86 |
| LLGMGEVVDANR | 0 | 1272.65 | 11013.71 | 19747.98 | 155.06 |
| MASTPYQLQFR | 0 | 1340.65 | 5811.20 | 38091.39 | 183.43 |
| MASTPYQLQFRK | 1 | 1468.75 | 2268.66 | 10011.79 | 110.66 |
| TNKYPR | 1 | 777.41 | 0.00 | 648.17 | 128.82 |
| WTPTLTKFEHR | 1 | 1414.74 | 0.00 | 644.79 | 44.85 |
| YVHGDVFR | 0 | 991.49 | 0.00 | 2203.03 | 75.85 |
|  |  |  | 22485.7 | 86270.4 |  |
